# Immediate weight bearing after dual orthogonal plate fixation of clavicle fractures: a propensity-matched retrospective cohort study

**DOI:** 10.1007/s00590-026-04959-9

**Published:** 2026-10-25

**Authors:** Andrew Monhollen, Lena Kolostov, Nash Kolb, Rown Parola, Anna Jacques, Wayne Hoskins, Douglas Haase

**Affiliations:** 1 Department of Orthopaedic Surgery, University of Missouri, Columbia, USA; 2 School of Medicine, University of Missouri, Columbia, USA; 3 Faculty of Medicine, Dentistry and Health Sciences, The University of Melbourne, Melbourne, Australia

**Keywords:** Clavicle fracture, Immediate weight-bearing, Patient reported outcomes, Dual-plate fixation

## Abstract

**Purpose:**

To evaluate whether immediate weight bearing as tolerated (WBAT) following open reduction and internal fixation of clavicle fractures with a dual mini-fragmentation plate construct increases complications, implant failure, or worsens functional and patient-reported outcomes compared to a restricted weight-bearing protocol.

**Methods:**

Adult patients (≥ 18 years) who underwent open reduction and internal fixation of a clavicle fracture were retrospectively identified and stratified into restricted weight-bearing or weight bearing as tolerated (WBAT) cohorts. Cohorts were matched for demographic and injury characteristics. All WBAT fractures were treated with dual orthogonal mini-fragmentation plate fixation, whereas restricted weight-bearing fractures were treated with either single- or dual-plate fixation. Outcomes included complications (infection, wound dehiscence, hardware prominence, implant failure, and loss of reduction) and PROMIS Physical Function and Pain Interference scores.

**Results:**

Among 378 initial patients, 93 were included after propensity score matching: 62 in the restricted weight-bearing group and 31 in the WBAT group. Complication rates were similarly infrequent between groups (all *p* ≥ 0.326), with no fixation failures or deep surgical site infections occurring in the WBAT group.

**Conclusions:**

Early weight bearing as tolerated may be safe following dual mini-fragmentation plate fixation of clavicle fractures, with comparable complication rates, union rates, and patient-reported outcomes to restricted weight-bearing protocols. These findings support the use of early mobilization in patients with rigid internal fixation constructs and may facilitate improved functional recovery and earlier return to activity.

## Introduction

Clavicle fractures are common injuries, with an incidence ranging from approximately 29 to 59 per 100,000 person-years, and are most frequently observed in younger, active male populations sustaining high-energy mechanisms [1, 2]. Although historically managed nonoperatively, increasing evidence has demonstrated improved early functional outcomes and lower nonunion rates with operative fixation of displaced fractures [3–9]. Consequently, open reduction and internal fixation (ORIF) has become increasingly utilized for displaced and comminuted fracture patterns [6, 10].

Single-plate fixation remains the conventional surgical technique; however, dual orthogonal mini-fragment plating has gained popularity due to its potential biomechanical and clinical advantages. Biomechanical studies have demonstrated that dual plating constructs provide comparable stability to traditional single precontoured plates across axial, bending, and torsional loading conditions [11–13]. More recent investigations suggest that certain dual plate configurations may even offer increased stiffness and resistance to failure while limiting interfragmentary motion [14]. Clinically, dual plate fixation has been associated with high union rates, decreased implant removal, and improved fixation of comminuted fracture patterns [13, 15, 16].

A key consideration following clavicle fracture fixation is postoperative rehabilitation, particularly early weight-bearing and mobilization. Early mobilization has well-established benefits in trauma patients, including improved functional recovery and decreased hospital length of stay [17, 18]. Despite this, concern persists that early weight-bearing following clavicle ORIF may increase the risk of fixation failure, loss of reduction, or complications.

Given the increasing utilization of dual plate constructs and their favorable biomechanical profile, early weight-bearing as tolerated (WBAT) may be feasible without compromising outcomes. However, clinical evidence evaluating this strategy remains limited.

The purpose of this study was to evaluate whether immediate WBAT following clavicle fracture ORIF increases complications, implant failure, or worsens patient-reported outcomes compared to restricted postoperative protocols. We hypothesized that WBAT following rigid fixation dual fixation constructs would result in comparable complication rates and functional outcomes.

## Methods

### Study design and setting

This retrospective cohort study was conducted at a single-center Level I academic trauma center following institutional review board approval. Adult patients who underwent open reduction and internal fixation (ORIF) of clavicle fractures between 2020 and 2025 were identified from institutional records. No a priori power analysis was performed; all eligible patients meeting inclusion criteria during the study period were included in the analysis.

### Patient selection

Patients aged ≥ 18 years with acute clavicle fractures (proximal, middle, or distal third) treated operatively were eligible for inclusion. Patients were followed until clinical or radiographic union. Clinical union was defined as resolution of pain at the fracture site, and radiographic union was defined by the presence of bridging callus. Patients who achieved clinical union were not routinely followed beyond that point for additional radiographic confirmation. Exclusion criteria included open fractures, pathologic fractures, revision fixation procedures, and nonunion repair cases. Patients with less than 6 weeks (42 days) of postoperative follow-up were excluded from the analysis. Polytrauma patients were included without restriction.

### Cohort definition and matching

Patients were stratified into two cohorts based on postoperative weight-bearing instructions: restricted weight bearing and WBAT. Propensity score matching was performed in a 2:1 ratio (restricted: WBAT) using age, sex, race, and concomitant injuries. All WBAT patients were treated by a single orthopedic trauma surgeon (DRH), while the restricted cohort included patients treated by multiple orthopedic trauma surgeons at a single institution.

### Surgical technique and postoperative protocols

**Fig. 1:**
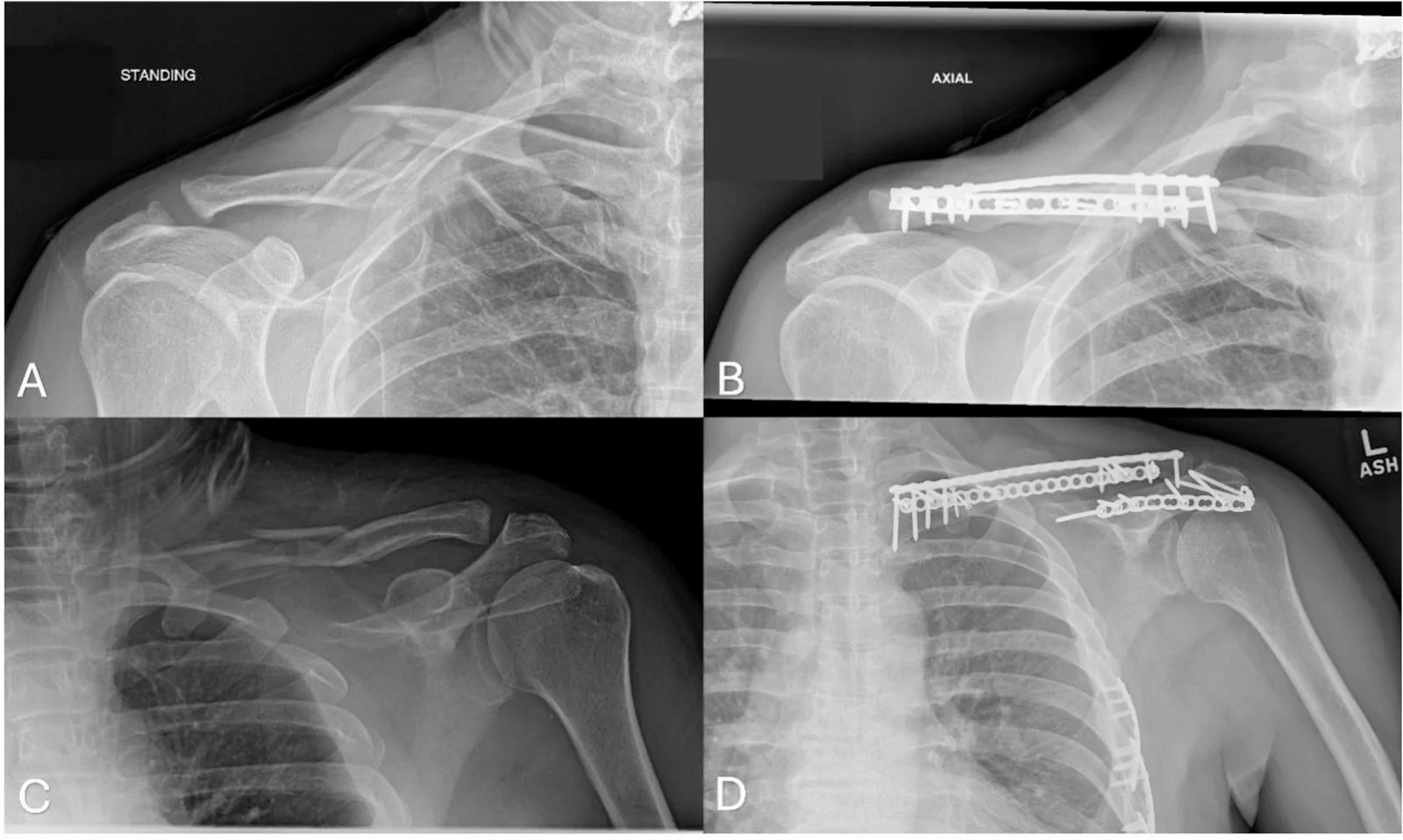
Anteroposterior (AP) radiographic images of an isolated, displaced right midshaft clavicle fracture preoperatively (**A**) and one year postoperative after dual orthogonal mini-fragmentation plate fixation (**B**) with successful healing following open reduction and internal fixation (ORIF) with immediate weightbearing as tolerated (WBAT). AP radiographic images of a comminuted left clavicle fracture with associated scapular spine and rib fractures preoperatively (**C**) and 1 year postoperative (**D**) with successful healing following ORIF with immediate WBAT

All patients underwent ORIF with construct selection at the discretion of the treating surgeon. The WBAT cohort was treated exclusively with dual orthogonal mini-fragmentation plating constructs (Fig. 1).

Patients in the WBAT cohort were instructed to use the operative extremity as tolerated, without formal restriction on weight bearing, range of motion, or lifting, Patients in the restricted cohort were limited to lifting no more than approximately 2 pounds (or equivalent to a light object such as a glass of water) and range of motion to shoulder height. Instructions were communicated to the patient or caregiver via post-operative instruction and/or discharge paperwork. For the restricted cohort, weight-bearing and activity restrictions were reassessed and updated at subsequent follow-up encounters as clinically appropriate.

### Data collection

Demographic variables included age, sex, race, tobacco use, alcohol use, illicit drug use, and comorbidities (diabetes and osteoporosis). Injury characteristics included mechanism of injury, fracture location, and OTA classification. Fracture location, OTA classification, and radiographic union were reviewed and confirmed by two fellowship trained orthopedic trauma surgeons.

Operative variables included number of plates, plate type, plate position, construct type (lag/neutralization plate, bridge plate, or compression plate), number of screws, cortices of fixation, and plate length (measured by number of holes).

### Outcomes

Postoperative outcomes included hardware prominence, wound dehiscence and/or superficial infection, deep surgical site infection, and fixation failure. Fixation failure was defined as loss of reduction and/or hardware failure, including plate or screw breakage, loosening, or need for revision fixation.

Patient-reported outcomes were assessed using PROMIS Physical Function and PROMIS Pain Interference at 6 weeks postoperatively.

### Statistical analysis

Continuous variables were assessed for normality and reported as means with standard deviations or medians with interquartile ranges, as appropriate. Normally distributed continuous variables were compared using Student’s t-tests, while non-normally distributed variables were compared using Mann-Whitney U tests. Categorical variables were reported as counts and percentages and compared using chi-square or Fisher’s exact test, as appropriate. Odds ratios (ORs) with 95% confidence intervals (CIs) were calculated for complication outcomes. Subgroup analyses were performed according to fracture location (proximal, middle, or distal third) and fixation construct (dual-plate fixation). Statistical significance was set at *p* < 0.05. All analyses were performed using RStudio (R Version 2026.07.1).

## Results

**Fig. 2:**
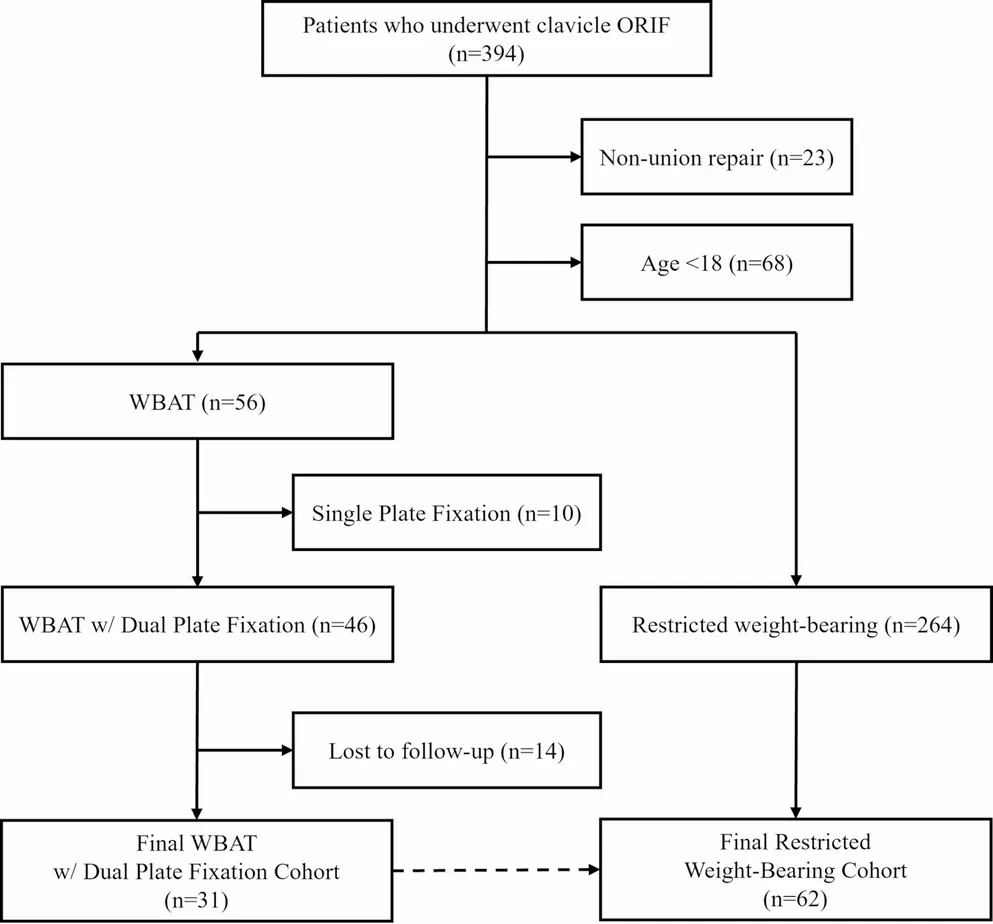
Consort diagram

A total of 378 patients were initially identified, with 93 patients included after matching. The final cohort consisted of 31 patients in the WBAT group and 62 patients in the restricted weight-bearing group (Fig. 2). The median follow-up for the WBAT group was 11.0 weeks (IQR 6.8–18.4) and the median follow-up for the restricted weight-bearing group was 24.7 (IQR 12.8–38.8).

There were no significant differences in baseline demographics between cohorts (Table 1).

**Table 1:** Baseline demographic, clinical, injury, and fracture characteristics of the WBAT and restricted weight-bearing cohorts following clavicle fracture fixation

	WBAT	Restricted WB	*p*
*n*	31	62	
Age	47.16	48.55	0.71
*Sex*			0.93
Male (n, %)	25	48 (77.4)	
Female (n, %)	6	14 (22.6)	
*Race*			0.86
White (n, %)	29 (93.5)	60 (96.7)	
Hispanic (n, %)	1 (3.2)	2 (3.2)	1.00
Tobacco use (n, %)	14 (45.2)	25 (40.3)	0.82
EtOH use (n, %)	13 (41.9)	25 (40.3)	1.00
Illicit drug use	3 (9.7)	2 (3.2)	0.42
Diabetes (n, %)	1 (3.2)	3 (4.8)	1.00
Osteoporosis	0 (0)	1 (1.6)	1.00
*Mechanism of injury*			0.76
MVC (n, %)	7 (22.6)	8 (12.9)	
MCC/ATV (n, %)	13 (41.9)	29 (46.8)	
Pedestrian versus Auto/train (n, %)	1 (3.2)	3 (4.8)	
Bike/scooter (n, %)	1 (3.2)	6 (9.7)	
Fall from height (n, %)	3 (9.7)	6 (9.7)	
Gound-level fall (n, %)	3 (9.7)	7 (11.3)	
Assault (n, %)	3 (9.7)	3 (4.8)	
*Fracture location*			0.53
Proximal 1/3 (n, %)	1 (3.2)	1 (1.6)	
Middle 1/3 (n, %)	25 (80.7)	45 (72.6)	
Distal 1/3 (n, %)	5 (16.1)	16 (25.8)	
*OTA fracture classification*			0.37
15.2 A (n, %)	18 (58.1)	29 (46.8)	
15.2 B (n, %)	11 (35.5)	20 (32.3)	
15.2 C (n, %)	2 (6.4)	13 (20.9)	
*Additional injuries*			0.19
None	14 (45.2)	31 (50.0)	
Shoulder/chest wall	9 (29.0)	7 (11.3)	
Upper extremity	1 (3.2)	2 (3.2)	
Axial	0 (0.0)	0 (0.0)	
Lower extremity	0 (0.0)	4 (6.5)	
Multiple	7 (22.6)	18 (29.0)	

WBAT, weight-bearing as tolerated; EtOH, alcohol use; MVC, motor vehicle collision; MCC, motorcycle collision; ATV, all-terrain vehicle; OTA, orthopaedic trauma association. Continuous variables are presented as mean values and categorical variables as number (%). P values represent comparisons between the WBAT and restricted weight-bearing cohorts

### Operative characteristics

Operative characteristics differed between cohorts. The WBAT group demonstrated uniform use of dual orthogonal plating constructs, whereas the restricted cohort included both single- and dual-plate constructs (*p* < 0.001). Implant selection varied significantly between groups (*p* = 0.001), with greater heterogeneity observed in the restricted cohort. Plate positioning also differed (*p* < 0.001); WBAT patients were treated exclusively with orthogonal constructs, while the restricted cohort included anterior, superior, and orthogonal configurations. Construct type did not differ significantly between groups (*p* = 0.137). WBAT patients demonstrated greater fixation density, with higher mean screw counts (*p* < 0.001) and greater cortices of fixation (*p* < 0.001) (Table 2). Plate length was similar between groups when stratified by implant type (Table 3).

**Table 2:** Operative fixation characteristics of the WBAT and restricted weight-bearing cohorts following clavicle fracture fixation

	WBAT	Restricted WB	*p*
*Number of plates*			< 0.001*
Single plate (n, %)	0 (0)	37 (59.7)	
Dual plate (n, %)	31 (100)	25 (40.3)	
*Plate*			0.001*
2.7-mm DCP/LCP (n, %)	39 (62.9)	54 (62.1)	
2.4-mm DCP/LCP (n, %)	23 (37.1)	16 (18.4)	
3.5-mm Pre-contoured (n, %)	0 (0)	8 (9.2)	
2.0-mm Mini fragment (n, %)	0 (0)	5 (5.7)	
Continuous compression implant (n, %)	0 (0)	4 (4.6)	
*Plate position*			< 0.001*
Anterior (n, %)	0 (0)	11 (17.7)	
Superior (n, %)	0 (0)	26 (41.9)	
Orthogonal (n, %)	31 (100)	25 (40.3)	
*Construct type*			0.137
Lag screw/neutralization (n, %)	23 (74.2)	35 (56.5)	
Compression (n, %)	7 (22.6)	17 (27.4)	
Bridge (n, %)	1 (3.2)	10 (16.1)	
Screws (mean ± sd)	14.48 ± 1.91	9.34 ± 3.04	< 0.001*
Cortices of fixation (mean ± sd)	28.65 ± 3.89	18.61 ± 5.99	< 0.001*

WBAT, weight-bearing as tolerated; DCP, dynamic compression plate; LCP, locking compression plate; SD, standard deviation. Continuous variables are presented as mean ± standard deviation and categorical variables as number (%). *Statistically significant (*P* < 0.05)

**Table 3:** Comparison of plate length by implant type between the WBAT and restricted weight-bearing cohorts following clavicle fracture fixation

	WBAT		Restricted WB		*p*
	*n*	# of holes (mean ± sd)	*n*	# of holes (mean ± sd)	
*2.7-mm DCP/LCP*	39	11.6 ± 2.7	54	11.2 ± 1.9	0.779
Proximal (1/3)	2	9 ± 1.4	2	12.5 ± 3.5	0.414
Middle (1/3)	31	12.0 ± 2.8	37	11.2 ± 1.8	0.672
Distal (1/3)	6	10.2 ± 1.0	15	10.9 ± 2.0	0.201
*2.4-mm DCP/LCP*	22	9.9 ± 2.6	16	9.5 ± 1.9	0.89
Proximal (1/3)	0	–	0	–	
Middle (1/3)	18	9.9 ± 2.7	14	9.3 ± 2.0	0.705
Distal (1/3)	4	9.5 ± 1.9	2	11 ± 0.0	0.475
*3.5-mm Pre-contoured*	0	–	8	8.9 ± 2	
Proximal (1/3)	0	–	0	–	
Middle (1/3)	0	–	5	8.4 ± 1.5	
Distal (1/3)	0	–	3	9.7 ± 2.9	
*2.0-mm Mini fragment*	0	–	5	5.2 ± 1.1	
Proximal (1/3)	0	–	0	-	
Middle (1/3)	0	–	3	4.7 ± 0.6	
Distal (1/3)	0	–	2	6.0 ± 1.4	

WBAT, weight-bearing as tolerated; DCP, dynamic compression plate; LCP, locking compression plate; SD, standard deviation. Plate length is reported as the mean number of plate holes ± standard deviation

### Complications

No patients in the immediate WBAT cohort experienced fixation failure. However, 2 patients (3.2%) experienced fixation failure in the restricted weightbearing cohort (Table 4).

**Table 4:** Clinical characteristics and management of fixation failure cases in the restricted weight-bearing cohort

Patient	Patient/injury characteristics	Index fixation	Complication details	Management	Outcome
Patient 1	Active smoker; motorcycle collision; AO/OTA 15.2C1 middle-third clavicle fracture	Dual plating with 12-hole 2.4-mm plate superiorly and 8-hole 2.7-mm plate anteriorly	At 8 weeks, fall from wheelchair resulted in reinjury and failure of the superior plate; fracture remained reasonably aligned. Intraoperative loosening of the anterior plate was noted at revision.	Revision ORIF with two 10-hole 2.7-mm plates; continued WB restrictions	Uneventful fracture union
Patient 2	Active smoker; motorcycle collision; AO/OTA 15.2C1 middle-third clavicle fracture	Single 12-hole 2.7-mm plate anteriorly	At 8 weeks, fracture displacement occurred following return to work and increased activity.	Revision ORIF with dual orthogonal plating using a 10-hole 2.4-mm superior plate and 12-hole 2.7-mm anterior plate; continued WB restrictions. Draining seroma developed 4 weeks after revision, requiring operative exploration; hardware removed 8 months after index procedure.	Cultures remained negative; ultimately achieved fracture union

Superficial wound complications occurred in 4 patients (6.5%) in the restricted cohort and 1 patient (3.2%) in the weight-bearing as tolerated cohort. All patients had closed injuries without local soft tissue compromise at the operative site and sustained high-energy mechanisms as part of polytrauma presentations. In the restricted cohort, 2 patients underwent hardware removal with wound closure for persistent wound dehiscence, 1 patient underwent superficial wound debridement with closure, and 1 patient was successfully treated with oral antibiotics alone. The single patient in the weight-bearing as tolerated cohort with a superficial wound complication was managed nonoperatively with oral antibiotics.

One patient in the restricted weight-bearing cohort developed a septic nonunion. The patient had an AO/OTA 15.2 C fracture, was a non-smoker, and sustained a high-energy mechanism of injury. The index fixation was performed using dual 2.7-mm plates (10-hole and 13-hole) in a bridge configuration with Ethibond cerclage augmentation. The patient had persistent fracture related pain and underwent hardware removal at 11 months postoperatively, at which time a fibrous nonunion was identified. At 14 months, the patient underwent nonunion repair with bone grafting. The postoperative course was complicated by *Pseudomonas* osteomyelitis requiring multiple irrigation and debridement procedures. At 3.5 years after the index procedure, the patient underwent revision nonunion repair and subsequently achieved fracture union without further intervention.

Overall, there were no significant differences between cohorts in complication rates (all *p* ≥ 0.326) (Table 5).

**Table 5:** Postoperative complications and fixation outcomes of the WBAT and restricted weight-bearing cohorts following clavicle fracture fixation

	WBAT	Restricted WB	*P*	OR [95% CI]
Hardware prominence (*n*, %)	2 (6.5)	10 (16.1)	0.325	2.76 [0.53–27.64]
*Fracture location*				
Proximal (1/3)	0	0		
Middle (1/3)	1	8	0.143	
Distal (1/3)	1	2	1.00	
*Number of plates*				
Dual plate	2	3	0.446	
Wound dehiscence and/or superficial infection (n, %)	1 (3.2)	3 (4.8)	1.00	
*Fracture location*				
Proximal (1/3)	0	0		
Middle (1/3)	0	3	0.548	
Distal (1/3)	1	0	0.238	
*Number of plates*				
Dual plate	1	2	0.581	
Deep surgical site infection (n, %)	0 (0)	1 (1.6)	1.00	
*Fracture location*				
Proximal (1/3)	0	0		
Middle (1/3)	0	1	1.00	
Distal (1/3)	0	0		
*Number of plates*				
Dual plate	0	1	0.446	
Total fixation failures (n, %)	0 (0)	2 (3.2)	0.551	
*Facture location*				
Proximal (1/3)	0	0		
Middle (1/3)	0	2	0.534	
Distal (1/3)	0	0		
*Number of plates*				
Dual plate	0	1	0.446	

WBAT, weight-bearing as tolerated; OR, odds ratio; CI, confidence interval. Categorical variables are presented as number (%). Odds ratios with 95% confidence intervals compare the restricted weight-bearing cohort with the WBAT cohort

### Patient reported outcome measures

Patient-reported outcomes were available for a subset of patients at 6-week follow-up (WBAT, *n* = 12 [61.3% missing]; restricted, *n* = 29 [53.2% missing]). No significant differences were observed in PROMIS Physical Function (*p* = 0.333), or PROMIS Pain Interference (*p* = 0.79) (Table 6).

**Table 6:** Comparison of patient-reported outcomes between the WBAT and restricted weight-bearing cohorts following clavicle fracture fixation

	WBAT (mean ± sd) *n* = 12	Restricted WB (mean ± sd) *n* = 29	Mean difference [95% CI]	*p*
PROMISphysical function	41.0 ± 13.0	36.8 ± 10.5	− 4.2 [− 4.7–13.2]	0.333
PROMIS pain interference	57.7 ± 9.6	56.9 ± 9.7	− 0.9 [− 6–7.7]	0.79

WBAT, weight-bearing as tolerated; PROMIS, patient-reported outcomes measurement information system; SD, standard deviation; CI, confidence interval. Data are presented as mean ± standard deviation. Mean differences are reported as the restricted weight-bearing cohort minus the WBAT cohort with corresponding 95% confidence intervals

## Discussion

In this retrospective cohort study, patients fixed with dual plate fixation and allowed unrestricted use of the operative extremity as tolerated were not associated with increased complications, fixation failure, or inferior early patient-reported outcomes. Complication rates were low and not significantly different between cohorts, including hardware prominence, wound complications, and fixation failure. Notably, no fixation failures occurred in the immediate weight-bearing cohort. Furthermore, early patient-reported outcomes at 6 weeks, including physical function and pain interference, were equivalent between groups. These findings suggested that early unrestricted use of the operative extremity did not negatively affect short-term clinical outcomes when stable fixation was achieved.

Clavicle fractures are common injuries, particularly in younger, active populations, and operative management has increased due to improved early outcomes and reduced nonunion rates compared with nonoperative treatment [1–4]. Among operative strategies, dual orthogonal plating has emerged as an alternative to traditional single-plate fixation.

Biomechanical studies have consistently demonstrated that dual mini-fragment plating constructs provide stability comparable to single anatomic plates under axial, torsional, and bending loads [11–13]. These constructs offer theoretical advantages through orthogonal plate orientation, improving resistance across multiple loading planes. More recent data suggest that certain dual plating configurations may provide increased stiffness, higher resistance to failure, and reduced interfragmentary motion, particularly in unstable fracture patterns [14]. However, construct performance is not uniform across implant sizes, as thinner constructs have demonstrated increased deformation and reduced fatigue resistance [19].

Clinically, dual plating has demonstrated excellent outcomes, with union rates approaching 100% and low complication rates [13, 15, 16]. In addition, dual plating has been associated with decreased implant removal rates, likely due to lower implant prominence [15]. Although concerns exist regarding increased soft tissue dissection and construct stiffness, these have not translated into increased nonunion or complication rates with appropriate surgical technique [15, 16].

Early mobilization following clavicle fixation has been evaluated in polytrauma patients with concomitant lower-extremity injuries requiring restricted weight bearing. One study found that immediate WBAT was associated with shorter hospital stays and improved physical therapy performance, while another demonstrated no increase in hardware failure or adverse effect on ultimate fracture union with early upper-extremity weight bearing [17, 18]. However, both studies focused on polytrauma patients with lower-extremity injuries, and neither directly evaluated the broader population of patients undergoing clavicle fixation with varying injury burdens. The present study extends this literature by directly comparing immediate unrestricted use of the operative extremity with postoperative restrictions in an inclusive cohort, while evaluating fixation failure, complications, and early functional outcomes. Additionally, all patients in the WBAT cohort underwent dual orthogonal plate fixation, providing a distinct perspective on immediate unrestricted use following stable fixation. The findings of the current study were consistent with prior biomechanical and clinical evidence and extend these observations specifically to early weight-bearing protocols in the setting of dual plating constructs. The absence of fixation failure in the weight-bearing cohort aligned with biomechanical literature demonstrating that dual orthogonal constructs provide sufficient rigidity to tolerate physiologic loading across multiple planes [11, 12]. The multidirectional stability inherent to orthogonal plate orientation likely mitigates the mechanical limitations observed in single-plate constructs under off-axis loading conditions, which is particularly relevant during early, unprotected use of the extremity [13].

An important consideration in interpreting these findings is the specific implant configuration used in the weight-bearing cohort, which consisted of a 2.7-mm superior plate combined with a 2.4-mm anteroinferior plate. Biomechanical studies evaluating dual plating constructs have demonstrated variability in performance based on plate thickness and construct composition. Pastor et al. reported that thinner 2.0/2.0-mm dual constructs exhibited increased deformation and reduced fatigue resistance despite similar stiffness, whereas thicker constructs (2.5/2.0-mm) demonstrated improved resistance to failure approaching that of traditional 3.5-mm plates [19]. Similarly, Prasarn et al. demonstrated that hybrid constructs incorporating a relatively thicker superior plate improved biomechanical performance under torsional and axial loading [13]. More recent work has further suggested that increased cross-sectional profile in dual constructs enhances resistance to failure and limits interfragmentary motion [14]. The 2.7/2.4-mm configuration used in the present study represents an intermediate-profile construct within this spectrum and likely achieves a balance between mechanical rigidity and implant profile. The absence of fixation failure with early weight bearing in this study suggests that this construct provides sufficient stability for early loading; however, these findings may not be generalizable to thinner dual plate configurations, and appropriate implant selection remains an important consideration.

The comparable complication rates and patient-reported outcomes observed in this study were consistent with prior clinical investigations demonstrating favorable results with both dual plating and early mobilization protocols [14, 16–18]. Increased fixation density in the weight-bearing cohort, including greater screw counts and cortices of fixation, may have further contributed to construct stability, as supported by prior biomechanical studies evaluating cyclic loading performance [14, 19]. Hardware-related complications were similarly comparable between cohorts and aligned with literature demonstrating favorable implant tolerance with low-profile dual constructs [15]. Although hardware removal for implant prominence remains a consideration, concerns regarding refracture after implant removal do not appear increased with dual plating constructs, as biomechanical studies have demonstrated no difference in load to failure compared with single plating [20]. The occurrence of a septic nonunion in a patient treated with dual 2.7-mm bridge plating highlights that mechanical stability alone does not ensure successful fracture healing, emphasizing the importance of biologic factors [13, 16]. Furthermore, excessive construct rigidity, particularly when combined with restricted postoperative loading, has been proposed to limit the beneficial effects of physiologic micromotion involved in secondary bone healing [14, 15]. Early weight bearing as tolerated may therefore provide a more favorable mechanical environment; however, this represents a potential mechanistic explanation rather than a finding directly evaluated in the present study.

This study has several limitations. Most importantly, the cohorts differed in fixation construct in addition to postoperative weight-bearing protocol. All WBAT patients underwent dual orthogonal plating, whereas the restricted cohort included single- and dual-plate constructs with variable implant configurations and fixation density. Thus outcomes cannot be attributed to WBAT alone, and the independent effect of fixation density could not be determined. Surgeon effect is another potential source of confounding, as all WBAT procedures were performed by a single surgeon, whereas the restricted cohort included multiple surgeons with potentially different techniques, implant selection, and reduction strategies. Although propensity score matching reduced baseline differences, important factors including facture patterns, surgeon, plate configuration, and fixation density were not included and may have contributed to residual confounding [21]. The relatively small cohort and low complication rate also limit the ability to detect differences in uncommon outcomes such as fixation failure, infection, or refracture. Adherence to prescribed postoperative weight-bearing and activity restrictions could not be reliably assessed due to inconsistent documentation in this retrospective cohort. Use of assistive devices was individualized based on concomitant injuries and functional needs, which may have resulted in variability in loading of the operative extremity within the restricted cohort and potentially influenced observed outcomes. Finally, PROMs were available for only a subset of patients, particularly in the WBAT cohort, and were limited to six-week follow-up, introducing potential follow-up bias and limiting assessment of long-term functional outcomes.

Early weight bearing as tolerated following open reduction and internal fixation of clavicle fractures with dual orthogonal fixation was not associated with increased complication rates, fixation failure, or worse early patient-reported outcomes compared with restricted postoperative protocols. These findings suggest that early unrestricted mobilization and weight bearing in patients treated with orthogonal dual fixation constructs used for clavicle fractures may be safe. Further investigation is warranted to draw definitive conclusions and refine evidence-based recommendations regarding postoperative rehabilitation protocols and construct selection in clavicle fixation.

## Data Availability

De-identified data supporting the findings of this study are available from the corresponding author on reasonable request, subject to institutional approval.

## Appendix

See Table 7.

**Table 7:** Baseline demographic characteristics of the excluded WBAT and restricted weight-bearing cohorts following propensity matching

	WBAT	Restricted WB	*p*
*n*	24	181	
Age	38.55	49.92	0.001
*Sex*			1.00
Male (n, %)	18 (75.0)	134 (64.0)	
Female (n, %)	6 (25.0)	47 (26.0)	
*Race*			0.783
White (n, %)	24 (100.0)	164 (93.2)	
Hispanic (n, %)	1 (4.2)	5 (2.8)	1.00
*Additional injuries*			0.098
None	7 (29.2)	82 (45.3)	
Shoulder/chest wall	6 (25.0)	26 (14.4)	
Upper extremity	0 (0.0)	1 (0.6)	
Axial	4 (16.7)	7 (3.9)	
Lower extremity	0 (0.0)	2 (1.1)	
Multiple	5 (20.8)	54 (29.8)	

WBAT, weight-bearing as tolerated; Continuous variables are presented as mean values and categorical variables as number (%). *P* values represent comparisons between the WBAT and restricted weight-bearing cohorts
